# Template-Free Synthesis of Monoclinic BiVO_4_ with Porous Structure and Its High Photocatalytic Activity

**DOI:** 10.3390/ma9080685

**Published:** 2016-08-11

**Authors:** Pengyu Dong, Xinguo Xi, Xinjiang Zhang, Guihua Hou, Rongfeng Guan

**Affiliations:** 1 Key Laboratory for Advanced Technology in Environmental Protection of Jiangsu Province, Yancheng Institute of Technology, Yancheng 224051, China; houguihua@ycit.cn (G.H.); Guanrongfeng@yahoo.com (R.G.); 2 Jiangsu Collaborative Innovation Center for Ecological Building Materials and Environmental Protection Equipments, Yancheng Institute of Technology, Yancheng 224051, China; xxg@ycit.cn; 3 School of Materials Engineering, Yancheng Institute of Technology, Yancheng 224051, China; zhangxj1983@yahoo.com

**Keywords:** bismuth vanadate, porous structure, photocatalysis

## Abstract

Monoclinic BiVO_4_ photocatalysts with porous structures were synthesized by a two-step approach without assistance of any templates. The as-prepared samples were characterized by X-ray diffraction pattern (XRD), scanning electron microscopy (SEM), Brunauer–Emmett–Teller (BET), ultraviolet–visible (UV–vis) diffuse reflectance spectroscopy (DRS), photocurrent responses, and electrochemical impedance spectra (EIS). It is found that the as-prepared BiVO_4_ samples had a porous structure with aperture diameter of 50–300 nm. Moreover, the BET specific surface area of the porous BiVO_4_-200 °C sample reaches up to 5.69 m^2^/g, which is much higher than that of the sample of BiVO_4_ particles without porous structure. Furthermore, a possible formation mechanism of BiVO_4_ with porous structure was proposed. With methylene blue (MB) as a model compound, the photocatalytic oxidation of organic contaminants in aqueous solution was investigated under visible light irradiation. It is found that the porous BiVO_4_-200 °C sample exhibits the best photocatalytic activity, and the photocatalytic rate constant is about three times of that of the sample of BiVO_4_ particles without porous structure. In addition, the photocurrent responses and electrochemical impedance spectra strongly support this conclusion.

## 1. Introduction

It is widely accepted that photocatalysis is a promising application technology in the fields of air cleaning and water purification [1]. However, the traditional TiO_2_ photocatalyst could not make use of visible light which accounts for 45% of solar spectrum due to a large band gap (3.2 eV). As a consequence, the development of new and efficient visible-light-driven photocatalysts attracts much attention.

BiVO_4_ is one of the typical complex oxides with narrow band-gap [2,3]. As an n-type semiconductor with a direct band gap of 2.4 eV [4], BiVO_4_ could absorb ample visible light and is stable, nontoxic, and relatively cheap [5]. It is generally considered that BiVO_4_ has three crystal systems of zircon-tetragonal, scheelite tetragonal, and scheelite-monoclinic phases [6]. However, only the monoclinic phase possesses an excellent visible-light photocatalytic property [7,8,9], which has been applied for degradation of various pollutants, e.g., organic dye [8,10], phenol [11], benzene [12], 4-n-alkylphenols [13,14], and so on. In past years, monoclinic BiVO_4_ with various morphologies, such as nanoparticles [15,16,17], nanorods [18], nanotubes [19], nanosheets [4,20], hierarchical nanostructure [21], has been successfully synthesized. Apart from these nanostructure morphologies, porous structures always show an improvement of the photocatalytic activity due to the large surface area and multiple scattering effects [22,23,24]. In recent years, mesoporous BiVO_4_ has been prepared by using templates or surfactants, such as silica (KIT-6) [25], silica aerogel [26], colloidal carbon spheres [27] as hard templates, and dodecylamine (DA), oleylamine (OL) or oleic acid (OA) as a surfactant [28], triblock copolymer P123 as a surfactant [11], and so on. Although these templates and surfactants could be removed by NaOH treatment or calcination process, it should be pointed out that the residual templates and surfactants may introduce impurities into the BiVO_4_ materials and will in any case complicate the separation process of photo-induced electrons and holes. Moreover, the additive of templates and surfactants may increase the cost. However, realization of controlled one-step template-free or surfactant-free fabrication of porous BiVO_4_ nanostructures remains a great challenge. Therefore, the development of a cheap and template-free method suitable for synthesis of high-purity porous BiVO_4_ structure is a significant research objective. Recently, Ying et al. reported a template-free approach to fabricate monoclinic BiVO_4_ hierarchical microtubes self-assembled by several nanowires [29]. Ma et al. prepared monoclinic BiVO_4_ hollow nanospheres via a template-free method using citric acid as chelating agent [30]. Cheng et al. fabricated monoclinic BiVO_4_ hollow microspheres by a one-pot template-free hydrothermal method [31]. Li et al. reported a hydrothermal method for synthesizing of monoclinic BiVO_4_ hollow microspheres from a novel vanadium source K_6_V_10_O_28_·9H_2_O without any additives but just through adjusting the reaction pH [32].

In this work, monoclinic BiVO_4_ with porous structure was synthesized through a novel two-step method without assistance of any templates. Compared with other template-free methods for preparing BiVO_4_ with hollow structure [29,30,31,32], our method is possibly not the best, but it provides a novel approach for the fabrication of monoclinic BiVO_4_ with porous structure by using the template-free approach. At the same time, the BiVO_4_ with porous structure prepared by our method exhibited a larger BET surface area compared with the previously reported BiVO_4_ hierarchical microtubes [29] and BiVO_4_ hollow microspheres [31]. Moreover, a possible formation mechanism of BiVO_4_ with porous structure was proposed. Furthermore, the photocatalytic performance of as-prepared monoclinic BiVO_4_ products with porous structure was evaluated by examining the degradation of methylene blue (MB) under visible light irradiation.

## 2. Experimental

### 2.1. Experimental Materials

The following analytically pure chemicals were used: bismuth nitrate (Bi(NO_3_)_3_·5H_2_O, Shanghai Qiangshun Chemical Reagent Co., Ltd., Shanghai, China), urea (Jiangsu Qiangsheng Chemical Co., Ltd., Changshu, China), ammonium metavanadate (NH_4_VO_3_, Shanghai Shanpu Chemical Co., Ltd., Shanghai, China), 25% ammonia solution (NH_3_·H_2_O, Shanghai pilot Chemical General Co., Shanghai, China), absolute ethanol (Jiangsu Tong Sheng Chemical Reagent Co., Ltd., Yixing, China), and MB dye (Tianjin Guangfu Fine Chemical Research Institute, Tianjin, China).

### 2.2. Synthesis

Firstly, the precursor was synthesized by a modified homogeneous precipitation method. Typically for the precursor, 0.002 mol of Bi(NO_3_)_3_·5H_2_O was dissolved into 30 mL of deionized water to form solution A, and 0.05 mol of urea was dissolved into 30 mL of deionized water to form solution B. Subsequently, solution B was added into solution A under stirring. After stirring for half an hour, the mixed solution was heated to 90 °C for 2 h in the water bath. The resulting suspension was separated by centrifugation and collected after washing with deionized water three times to obtain the precursor. Secondly, BiVO_4_ with porous structure was synthesized as follows: the as-obtained precursor was dispersed into 10 mL of deionized water by ultrasonication. Then, 0.002 mol of NH_4_VO_3_ was dissolved in deionized water (10 mL) with stirring and heating, and dripped into the above precursor suspension followed by further stirring at 70 °C for half an hour in the water bath. After cooling to room temperature, the final pH value was adjusted to be about 7.5 by ammonia solution. Afterwards, the suspension was transferred into a Teflon-lined autoclave of 50 mL capacity, and kept at various temperatures (i.e., 180, 200, 220 °C) for 12 h followed by cooling to ambient temperature naturally, respectively. The precipitate was separated from the reaction media by centrifugation, and then washed with deionized water and ethanol several times, and finally dried overnight at 80 °C to obtain the BiVO_4_ product with porous structure. For comparison, BiVO_4_ particles without porous structure were prepared as follows: 0.002 mol of Bi(NO_3_)_3_·5H_2_O was dissolved into 30 mL of deionized water to form solution A. Then, 0.002 mol of NH_4_VO_3_ was dissolved in deionized water (10 mL) with stirring and heating, and dripped into the solution A followed by further stirring at 70 °C for half an hour in the water bath, and the final pH value of the suspension was adjusted to be about 7.5 after cooling to room temperature. Subsequently, the suspension was transferred into a Teflon-lined autoclave and kept at 200 °C for 12 h followed by cooling to ambient temperature naturally. The precipitate was separated by centrifugation, and then washed with deionized water and ethanol several times, and finally dried overnight at 80 °C to obtain the sample of BiVO_4_ particles.

### 2.3. Characterization

X-ray diffraction (XRD) measurement was carried out with a D/max-2400 diffractometer (Rigaku, Tokyo, Japan) using Cu–Ka radiation. The morphologies of samples were examined by scanning electron microscopy (SEM, Hitachi S-4800, Hitachi High-Technologies Corp., Tokyo, Japan). The Brunauer-Emmett-Teller (BET) specific surface area and Barrett-Joyner-Halenda (BJH) pore distribution of the samples were measured using a Micromeritics ASAP 2020-M instrument (Micromeritics Instrument Corp., Norcross, GA, USA). Ultraviolet-visible diffuse reflectance spectra (UV-vis DRS) were measured using a Perkin Elmer 950 spectrometer (Perkin Elmer, Norwalk, CT, USA), while BaSO_4_ was used as a reference. The photocurrent response and electrochemical impedance spectra of as-prepared photocatalysts was measured on an electrochemical analyzer (CHI660E) (Shanghai Chenhua Instruments Co., Ltd., Shanghai, China) in a standard three-compartment cell using 0.5 M Na_2_SO_4_ (pH = 6.8) solution as the electrolyte. For the preparation of working electrodes for electrochemical measurements, a homogeneous catalyst ink was first prepared by dispersing 4 mg of catalyst material and 80 μL of a 5 wt % Nafion solution in 2 mL of H_2_O by ultrasonication, and then 400 μL of catalyst ink dispersions was drop-coated directly onto the precleaned indium tin oxide (ITO) glass surface by microsyringe and placed on a hot plate to speed drying. The surface of working electrode exposed to the electrolyte was a circular film with the geometrical surface areas of 4 cm^2^. Platinum foil was used as counter electrode and Ag/AgCl electrode as the reference electrode. A 350 W Xe lamp with a cutoff filter of 420 nm was used for excitation. The photo-responses of the photocatalysts as light on and off were measured at 0.0 V, and the electrochemical impedance spectra were measured at 0.0 V. A sinusoidal ac perturbation of 5 mV was applied to the electrode over the frequency range of 10–10^4^ Hz.

### 2.4. Evaluation of Photocatalytic Activity

The photocatalytic performance of as-prepared samples was evaluated by examining the degradation of MB under visible light irradiation. In a typical photocatalytic degradation process, 20 mg of photocatalyst was suspended in the MB solution (10 mg/L, 90 mL). Before irradiation, the suspensions were stirred magnetically in the dark for 60 min to ensure the establishment of adsorption–desorption equilibrium. A 350 W Xe lamp with a cutoff filter of 420 nm was employed for the visible-light irradiation source and positioned 17 cm away from the reactor to start the photocatalytic reaction under continuous stirring conditions. A certain volume of suspension was withdrawn at selected times for analysis. After recovering the photocatalyst by centrifugation (at 15,000 rpm for 10 min), the concentration of MB solution at some points was analyzed by measuring the light absorption of clear MB solution at 664 nm using a spectrophotometer (UV-2450; Shimadzu, Kyoto, Japan). The percentage of degradation was calculated by C/C_0_. Here, C is the concentration of remaining MB solution at each irradiated time interval, while C_0_ is the initial concentration.

## 3. Results and Discussion

### 3.1. XRD Analysis

**Figure 1:**
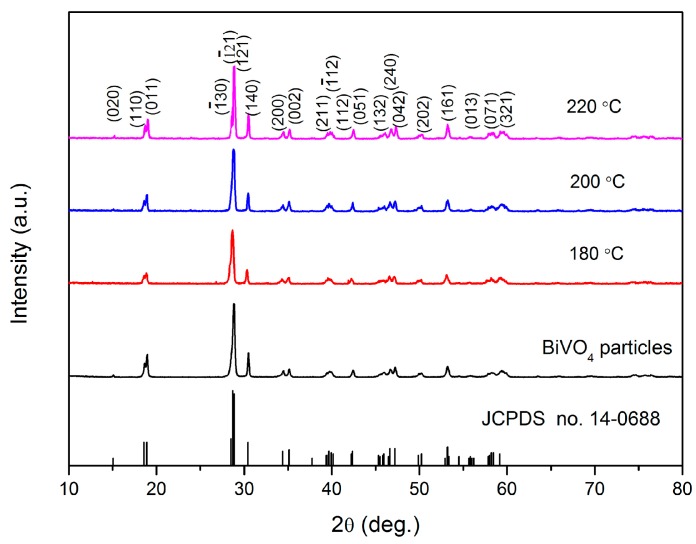
XRD patterns of BiVO_4_ particles and BiVO_4_ samples prepared at various hydrothermal temperatures.

The phase characteristics of the precursor were studied by XRD, as shown in Figure S1 in the Supplementary Materials. The diffraction peaks of the precursor can be indexed as [Bi_6_O_5_(OH)_3_](NO_3_)_5_·3H_2_O, agreeing well with the JCPD Standard card no. 48-0575. Based on the [Bi_6_O_5_(OH)_3_](NO_3_)_5_·3H_2_O precursor, BiVO_4_ products was synthesized. From Figure 1, it can be seen that all of the XRD patterns of BiVO_4_ products prepared at hydrothermal temperatures of 180, 200, and 220 °C are in good agreement with the standard data of monoclinic BiVO_4_ (JCPDS no. 14-0688), with no other characteristic peaks observed. This indicates that monoclinic BiVO_4_ products with well-crystallized high-purity were successfully obtained via the two-step processes. Moreover, it is found that monoclinic phase was achieved in the sample of BiVO_4_ particles without porous structure.

### 3.2. SEM Analysis

**Figure 2:**
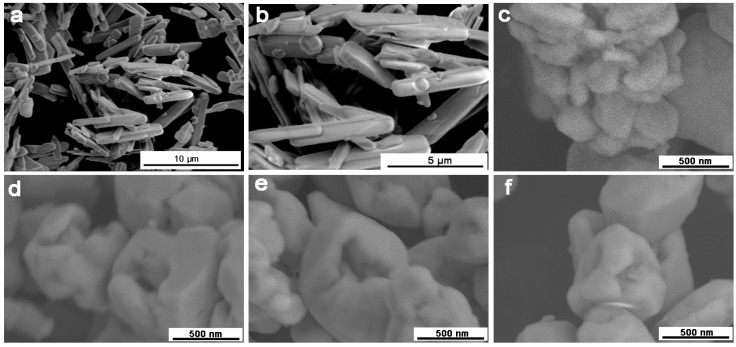
SEM images of the precursor (**a**,**b**); BiVO_4_ particles (**c**); and as-obtained BiVO_4_ products prepared at the hydrothermal temperatures of 180 °C (**d**); 200 °C (**e**); and 220 °C (**f**).

From Figure 2a,b, it is observed that the precursor displays a plate-like morphology. Moreover, the as-prepared sample of BiVO_4_ particles shows particle morphology with particle size of 200–500 nm (Figure 2c). Furthermore, the low magnification SEM image of as-obtained BiVO_4_ products prepared at the hydrothermal temperatures of 180 °C is presented in Figure S2 in the Supplementary Materials. It could be seen that the obtained BiVO_4_ product shows a porous structure. Large magnification SEM images of BiVO_4_ products prepared at the hydrothermal temperatures of 180 °C (Figure 2d), 200 °C (Figure 2e), and 220 °C (Figure 2f) reveal that the all of the BiVO_4_ products exhibit a porous structure composed of nanoflakes with thicknesses of 50–100 nm, which means that BiVO_4_ with porous structure was successfully achieved by our two-step method. Further observation indicates that the aperture diameter of the BiVO_4_ with porous structure is about 50–300 nm, which is near that of previously reported BiVO_4_ hollow nanospheres (the cavity diameter of 40 nm ) [30] and much smaller than that of BiVO_4_ hierarchical microtubes (the outer diameter of 1.2–1.5 μm) [29] and BiVO_4_ hollow microspheres (the diameter of 2–4 μm). [31].

### 3.3. Formation Mechanism

**Scheme 1:**
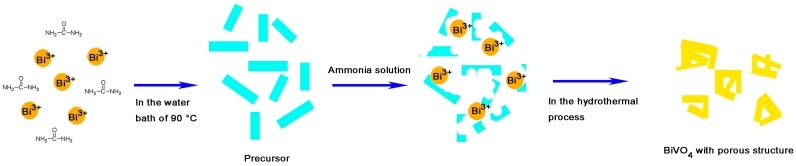
Schematic illustration for the possible formation mechanism of BiVO_4_ with porous structure.

A possible formation mechanism of as-obtained BiVO_4_ with porous structure was proposed, as presented in Scheme 1. Firstly, the [Bi_6_O_5_(OH)_3_](NO_3_)_5_·3H_2_O precursor with a plate-like morphology was synthesized in the water bath of 90 °C by a modified homogeneous precipitation method using Bi(NO_3_)_3_·5H_2_O as the raw material, deionized water as the solvent, and urea as the precipitating agent. Then, NH_4_VO_3_ was dissolved in deionized water and the pH value was adjusted to be about 7.5 by ammonia solution. In this process, VO_3_^−^ might react with OH^−^ resulting from ammonia solution to produce VO_4_^3−^ and H^+^ according to Equation (1). Subsequently, the H^+^ started to erode the plate-like [Bi_6_O_5_(OH)_3_](NO_3_)_5_·3H_2_O precursor from the surface gradually, giving rise to a large amount of Bi^3+^ according to Equation (2), which then reacted with the surrounding VO_4_^3−^ to produce a thin layer of BiVO_4_ on the surface of the plate-like [Bi_6_O_5_(OH)_3_](NO_3_)_5_·3H_2_O precursor (Equation (3)). Finally, in the hydrothermal process, the reaction with a fast diffusion rate of Bi^3+^ inside this BiVO_4_ layer and the slow diffusion rate of VO_4_^3−^ outside the BiVO_4_ layer led to the formation of porous structure. This explanation was called the Kirkendall effect, which deals with the movement of the interface between diffusion couples [33]. A similar Kirkendall phenomenon was reported in the preparation of YVO_4_ product [34].

VO_3_^−^ + OH^−^ → VO_4_^3−^ + H^+^ (1)

[Bi_6_O_5_(OH)_3_](NO_3_)_5_·3H_2_O + 13H^+^ → 6Bi^3+^ + 11H_2_O + 5NO_3_^−^ (2)

Bi^3+^ + VO_4_^3−^ → BiVO_4_ (3)

### 3.4. Nitrogen Adsorption–Desorption

**Figure 3:**
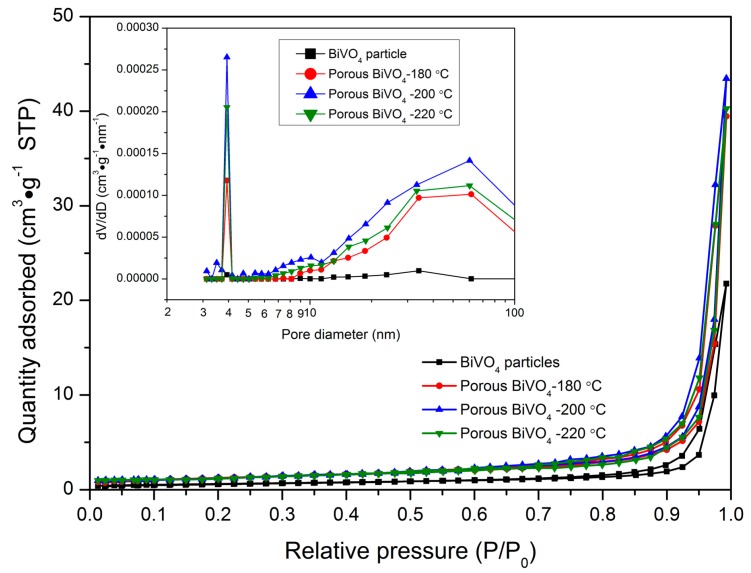
N_2_ adsorption-desorption isotherms of BiVO_4_ particles and BiVO_4_ samples prepared at various hydrothermal temperatures. The inset is the corresponding BJH pore size distributions.

The porous structure was further confirmed and characterized as follows. Figure 3 shows the nitrogen adsorption–desorption isotherms of BiVO_4_ particles and BiVO_4_ samples prepared at various hydrothermal temperatures. It can be seen that all the as-prepared samples show a type H3 hysteresis loop according to IUPAC classification [35], indicating the presence of slit-shaped pores or voids in the aggregates of plate like particles, which is consistent with the SEM results in Figure 2. Furthermore, the observed hysteresis loops of the as-prepared samples approach *P*/*P*_0_ = 1, suggesting the presence of macropores (>50 nm) [36]. These results suggest that pores are presented in the samples. The inset of Figure 3 shows the corresponding BJH pore size distributions of these samples. It is observed that the porous BiVO_4_ samples display two peaks around 4 and 60 nm, which could be attributed to the mesopores and macropores, respectively. On the contrary, there is no peak of pore size distribution for the sample of BiVO_4_ particles, suggesting there is no apparent pore. Table 1 shows the BET specific surface area and BJH pore volume of these samples. It can be seen that the porous BiVO_4_-200 °C sample shows the largest BJH pore volume and BET specific surface area (5.69 m^2^/g), which is much higher than that of the sample of BiVO_4_ particles without porous structure (1.85 m^2^/g). Moreover, it is found that the BET specific surface area of porous BiVO_4_-200 °C sample is much higher than that of the previously reported BiVO_4_ hierarchical microtubes (0.3 m^2^/g) [29] as well as BiVO_4_ hollow microspheres (1.05 m^2^/g) [31] and is near that of BiVO_4_ hollow spheres (5.85 m^2^/g) prepared by employing colloidal carbon spheres (CCSs) as hard templates [27]. The higher BET specific surface area is beneficial for the photocatalytic performance. In addition, it is noted that the porous BiVO_4_-220 °C sample exhibits smaller BET specific surface area compared with porous BiVO_4_-200 °C sample, which may be due to the higher hydrothermal temperature (i.e., 220 °C) inducing the collapse of porous structures.

### 3.5. UV-Vis Diffuse Reflectance Spectroscopy

**Figure 4:**
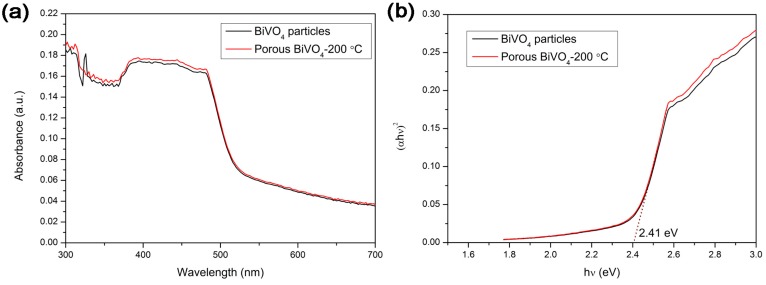
UV–vis diffuse reflection absorption spectra of the sample of BiVO_4_ particles as well as the porous BiVO_4_ sample prepared at the hydrothermal temperatures of 200 °C (**a**); and the plots of (*αhv*)^2^ versus photon energy *hv* of these two samples (**b**).

The optical absorption property is a key factor controlling the photocatalytic activity of a catalyst. Figure 4a shows the comparison of UV-vis absorption spectra of the sample of BiVO_4_ particles and porous BiVO_4_-200 °C sample. It is obvious that both of these samples exhibit strong absorption in the visible light range. The band gap energy (*Eg*) of these two samples was calculated from the absorption data using Equation (4) [37]:

(4) $$\alpha hv=A{\left(hv-Eg\right)}^{n}$$

where *α* is absorption coefficient; *A* is the absorption constant and *n* is a constant which depends on the probability of transition; it takes the values 1/2 and 2 for direct allowed and indirect allowed transitions, respectively. Because BiVO_4_ has an direct band-gap [38], the energy gap (*Eg*) of the samples was estimated from the intercept of the tangent in the plots of (*αhv*)^2^ versus photon energy *hv*. The calculated Eg values of both samples of BiVO_4_ particles and porous BiVO_4_ are 2.41 eV, as shown in Figure 4b. This result indicates that both BiVO_4_ particles and porous BiVO_4_ sample possess the same band gap, suggesting the morphology has little or no effect on the band gap of as-prepared samples.

### 3.6. Photocatalytic Activity and Electrochemical Analysis

**Figure 5:**
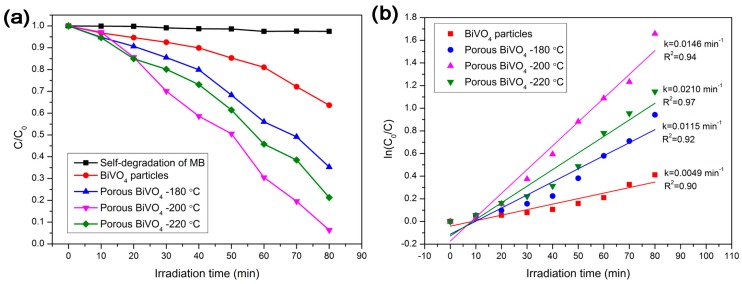
Photocatalytic degradation of MB solution (**a**) and plots of ln(C_0_/C) versus irradiation time (**b**) in the presence of as-prepared samples.

Before the photocatalytic reaction, the sample suspension was stirred for 1 h in the dark to reach the adsorption–desorption equilibrium. The adsorption isotherms of MB solutions for as-prepared samples are shown in Figure S3. It is obvious that the porous BiVO_4_-200 °C sample exhibits higher adsorption ability than other samples, which is in good agreement with the BET specific surface area. The photocatalytic behaviors of these as-prepared samples were then studied for degradation of MB solutions under visible light irradiation at room temperature. As shown in Figure 5a, the photocatalytic degradation efficiency of MB solutions follows the order: porous BiVO_4_-200 °C > porous BiVO_4_-220 °C > porous BiVO_4_-180 °C > BiVO_4_ particles. The variation of absorption intensity of MB solutions over these samples at different irradiation times is recorded (Figure S4), which strongly supports the above result. Additionally, it is known that the conduction band (E_CB_) edge and valence band (E_VB_) edge of BiVO_4_ were about 0.42 eV and 2.6 eV, respectively, which results in a weak reduction ability and high oxidation ability [31]. Therefore, it is generally considered that the mineralization and degradation of MB were ascribed to the high oxidation ability of BiVO_4_ [11,30,39]. One of the reasons for higher photocatalytic activity of porous BiVO_4_ samples is the higher BET specific surface area. It is generally considered that the photocatalytic reactions are typically surface-based processes and the photocatalytic efficiency is closely related to the adsorption property of dyes on the surfaces of a photocatalyst, which largely depends on the BET specific surface area. So, the higher the BET specific surface area, the stronger the adsorption of dyes on the photocatalyst surface, and thus the easier and faster the photocatalytic process [40]. Furthermore, the kinetics of photocatalytic reactions could be described using a first order reaction for low concentrations of MB solutions. The apparent rate constants (k, min^−1^) are displayed in Figure 5b, which is determined from the slopes of ln(C_0_/C) versus irradiation time. Remarkably, the photocatalytic rate constant of porous BiVO_4_-200 °C is about three times of that of the sample of BiVO_4_ particles for degradation of MB dye solutions. In addition, the correlation coefficient (R^2^) represents the correlation degree between practical photocatalytic reaction and linear fit. It is found that all of the R^2^ values are above 0.9, suggesting the data fit the first kinetic model well.

**Figure 6:**
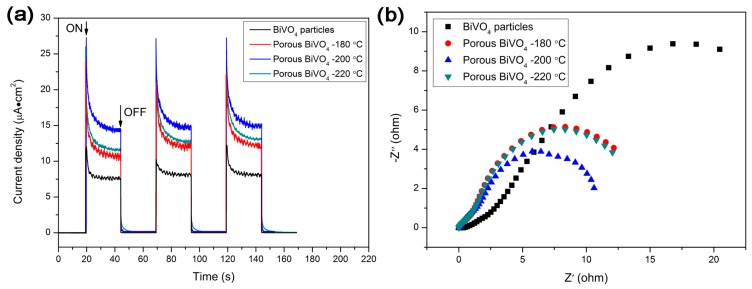
The photo-responses of as-prepared samples under visible light (**a**) and electrochemical impedance spectra (**b**) of these samples.

Figure 6a shows the transient photocurrent responses via three ON−OFF cycles that the electrodes deposited under visible light irradiation, which are correlated with the recombination efficiency of the photogenerated carriers [41,42,43]. It is found that the photocurrent efficiency is consistent with the photocatalytic activities, suggesting the separation and transfer of photoinduced electron−hole pairs are more efficient in the case of BiVO_4_-200 °C. The electrochemical impedance spectra provide further evidence, as shown in Figure 6b. It can been seen that the diameter of the arc radius for the BiVO_4_-200 °C electrode is smaller than that for the other electrodes, demonstrating it shows an enhanced separation and transfer efficiency of photogenerated e–h pairs because the smaller arc radius implies a higher efficiency of charge transfer.

Therefore, it can be concluded that the high BET specific surface area and effective separation transfer efficiency of photogenerated e–h pairs are the main reasons for the enhanced photocatalytic activity of as-prepared porous BiVO_4_ samples.

## 4. Conclusions

In summary, monoclinic BiVO_4_ photocatalysts with porous structures were successfully prepared by a two-step approach without assistance of any templates. Kirkendall effect was used to explain the formation mechanism of as-obtained BiVO_4_ with porous structure. Compared with the sample of BiVO_4_ particles without porous structure, the porous BiVO_4_ samples exhibit enhanced photocatalytic activity. In particular, the photocatalytic rate constant of porous BiVO_4_-200 °C sample could reach up to three times of that of the sample of BiVO_4_ particles. Furthermore, the high BET specific surface area and effective separation transfer efficiency of photogenerated e–h pairs are considered the main reason for the enhanced photocatalytic activity of as-prepared porous BiVO_4_ samples. Finally, it is anticipated that this synthesis method might be used to prepare other vanadate materials.

## Figures and Tables

**Table 1:** BET specific surface area and BJH pore volume of all of the as-prepared samples.

Samples	BiVO_4_ Particles	Porous BiVO_4_-180 °C	Porous BiVO_4_-200 °C	Porous BiVO_4_-220 °C
BET specific surface area (m^2^/g) ^1^	1.85	4.42	5.69	4.83
BJH pore volume (cm^3^/g) ^2^	0.023	0.055	0.072	0.061

^1^ Calculated from the linear part of the BET plot (P/P_0_ = 0.05–0.3); ^2^ Calculated from nitrogen desorption branch using the BJH algorithm.

## Supplementary Materials

The following are available online at www.mdpi.com/1996-1944/9/8/685/s1. Figure S1: XRD pattern of the precursor, Figure S2: Low magnification SEM image of as-obtained BiVO_4_ products prepared at the hydrothermal temperatures of 180 °C, Figure S3: The adsorption isotherms of MB in the presence of as-prepared samples under dark conditions, Figure S4: UV–vis absorption spectra of MB solutions separated from the photocatalysts of BiVO_4_ particles (a); and as-obtained BiVO_4_ products prepared at the hydrothermal temperatures of 180 °C (b); 200 °C (c); and 220 °C (d) suspensions during illumination.
