# Supplementary material for: Template-Free Synthesis of Monoclinic BiVO4 with Porous Structure and Its High Photocatalytic Activity
Source: Materials (Basel). 2016 Aug 11;9(8):685. doi: 10.3390/ma9080685 (PMC5512351; doi:10.3390/ma9080685)
Supplement: Supplementary file 1 [file materials-09-00685-s001.pdf]

Supplementary Materials: Template-Free Synthesis of Monoclinic $BiVO_4$ with Porous Structure and Its High Photocatalytic Activity

Pengyu Dong, Xinguo Xi, Xinjiang Zhang, Guihua Hou and Rongfeng Guan

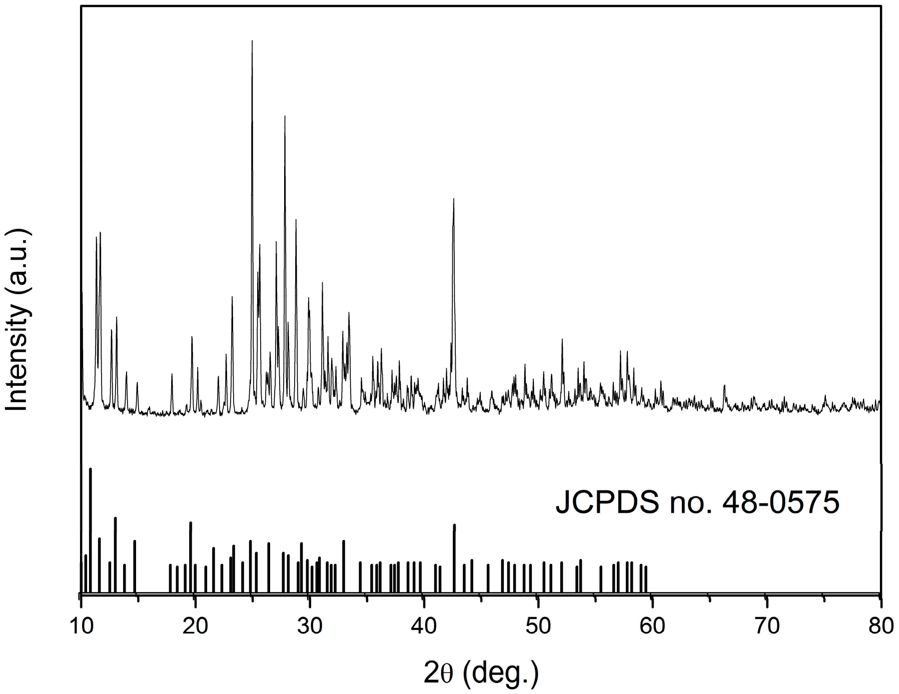

Figure S1. XRD pattern of the precursor.

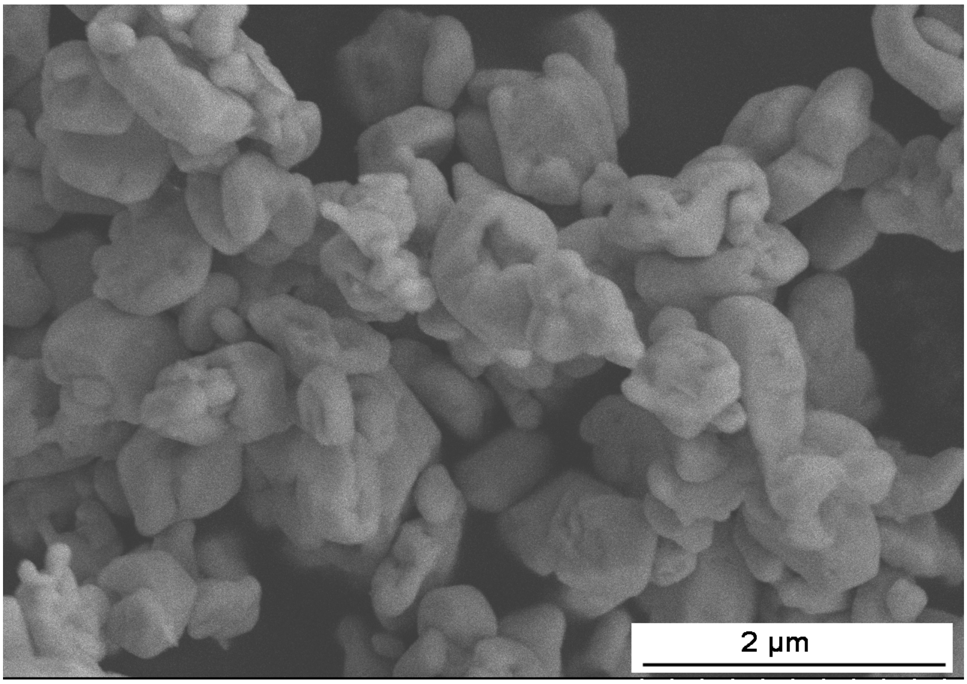

Figure S2. Low magnification SEM image of as-obtained $BiVO_4$ products prepared at the hydrothermal temperatures of 180 °C.

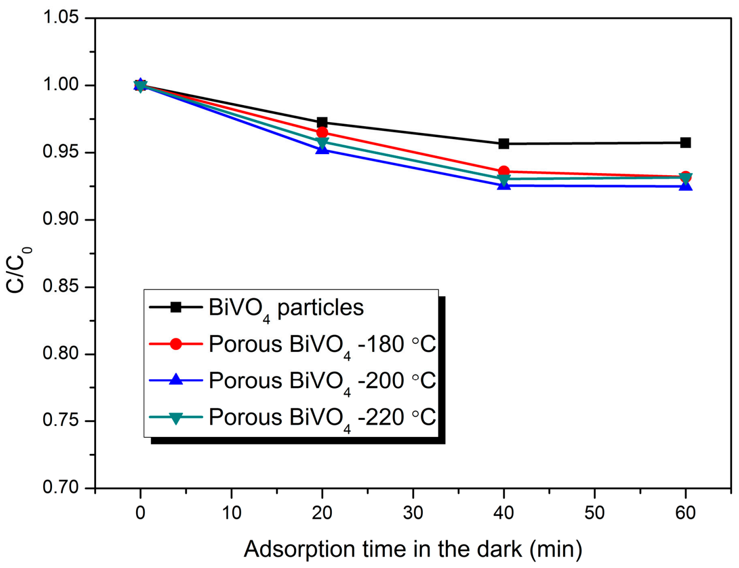

Figure S3. The adsorption isotherms of MB in the presence of as-prepared samples under dark conditions.

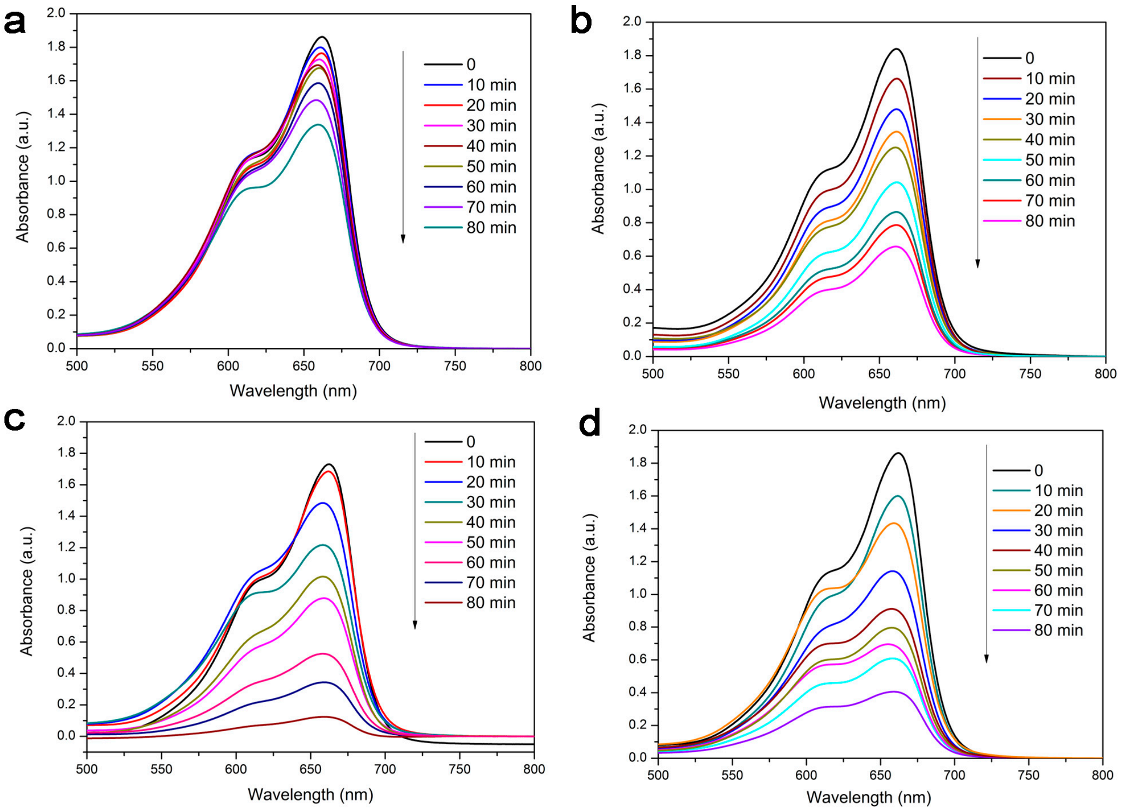

Figure S4. UV–vis absorption spectra of MB solutions separated from the photocatalysts of $BiVO_4$ particles (**a**); and as-obtained $BiVO_4$ products prepared at the hydrothermal temperatures of 180 °C (**b**); 200 °C (**c**); and 220 °C (**d**) suspensions during illumination.
